# A novel clinical perspective on subarachnoid hemorrhage complicated by myocardial injury: independent predictors and short-term prognostic assessment

**DOI:** 10.3389/fneur.2026.1908686

**Published:** 2026-07-29

**Authors:** Peng Xue, Yu Xin Jiang, Yi Guo, Hai Quan Li, Da He Han, Xiao Yan Meng, Yu Jie Li

**Affiliations:** 1 Department of ICU, The Second Affiliated Hospital of Xuzhou Medical University, Xuzhou, Jiangsu, China; 2 Department of RICU, The Second Affiliated Hospital of Xuzhou Medical University, Xuzhou, Jiangsu, China

**Keywords:** Glasgow Coma Scale, Glasgow Outcome Score, myocardial injury, neurogenic pulmonary edema, right optic nerve sheath diameter, subarachnoid hemorrhage

## Abstract

**Objective:**

To identify clinical, imaging, and laboratory factors associated with myocardial injury in patients with aneurysmal subarachnoid hemorrhage (aSAH) and to evaluate factors associated with short-term neurological outcomes at hospital discharge.

**Methods:**

In this single-center retrospective cohort study, clinical, laboratory, and imaging data were collected from 208 patients with aSAH admitted between 2022 and 2024. Univariable and multivariable logistic regression analyses, supported by Firth penalized logistic regression sensitivity analyses, were used to identify variables associated with myocardial injury and short-term neurological prognosis.

**Results:**

Right optic nerve sheath diameter (RONSD), procalcitonin (PCT), and neurogenic pulmonary edema (NPE) were independently associated with myocardial injury (adjusted ORs: 4.20, 1.45, and 18.87, respectively). Lower high-sensitivity C-reactive protein (hsCRP) levels, higher Glasgow Coma Scale (GCS) scores, younger age, and absence of hydrocephalus were associated with better short-term prognosis. Myocardial injury was not independently associated with poor short-term prognosis after adjustment.

**Conclusion:**

RONSD, PCT, and NPE may help identify patients with aSAH who are at increased risk of biomarker-defined myocardial injury. Short-term prognosis appears to be more strongly associated with inflammatory status, neurological impairment, age, and hydrocephalus than with myocardial injury alone. These single-center nomogram models should be considered preliminary and require external validation before routine clinical implementation.

## Introduction

Spontaneous subarachnoid hemorrhage (SAH) is primarily an acute neurovascular event caused by rupture of an intracranial aneurysm (1, 2). It is the third most common type of stroke, occurs more frequently in women and older individuals (3, 4), and remains associated with high early mortality and long-term neurological disability (5, 6). The pathophysiology of SAH is not confined to the intracranial compartment; it can also trigger systemic stress responses, neuroendocrine dysregulation, systemic inflammation, and multiorgan dysfunction. Myocardial injury and neurogenic pulmonary edema (NPE) are among the serious extracranial complications observed after SAH. Their mechanisms are not fully understood, but sympathetic overactivation, catecholamine-mediated calcium overload in cardiomyocytes, and coronary microcirculatory dysfunction are considered important contributors (5–11). Cardiac involvement after SAH may manifest as electrocardiographic abnormalities, including QT-interval prolongation, ST-segment depression, T-wave inversion, ventricular or supraventricular arrhythmias, or regional left ventricular wall-motion abnormalities (12–14). High-sensitivity cardiac troponin T (hs-cTnT) is a specific biomarker of myocardial injury and may support early detection and severity stratification (15, 16). Previous studies have reported troponin elevation in approximately 20–50% of patients with SAH (12, 17, 18), and troponin levels may correlate with clinical grading systems such as the Hunt-Hess and modified Fisher classifications (19, 20). However, whether myocardial injury independently affects short-term prognosis in SAH remains controversial. Therefore, early identification of factors associated with myocardial injury and short-term prognosis may support risk stratification and guide timely clinical management.

The occurrence and prognostic assessment of myocardial injury in patients with subarachnoid hemorrhage (SAH) have traditionally relied on factors such as the Glasgow Coma Scale (GCS) score, patient age, and Fisher grade (21). However, the mechanisms underlying systemic complications, including myocardial injury, neurogenic pulmonary edema (NPE), and inflammatory responses, remain incompletely understood. In this study, we investigated myocardial injury in 208 patients with aneurysmal SAH and its association with prognosis at discharge. The study focused on the relationships among clinical indicators, neurological injury, imaging findings, and laboratory parameters to provide a more comprehensive basis for early risk stratification. Given the retrospective single-center design, the proposed risk models are intended for exploratory bedside risk stratification and future validation rather than immediate routine clinical implementation.

## Materials and methods

**Figure 1:**
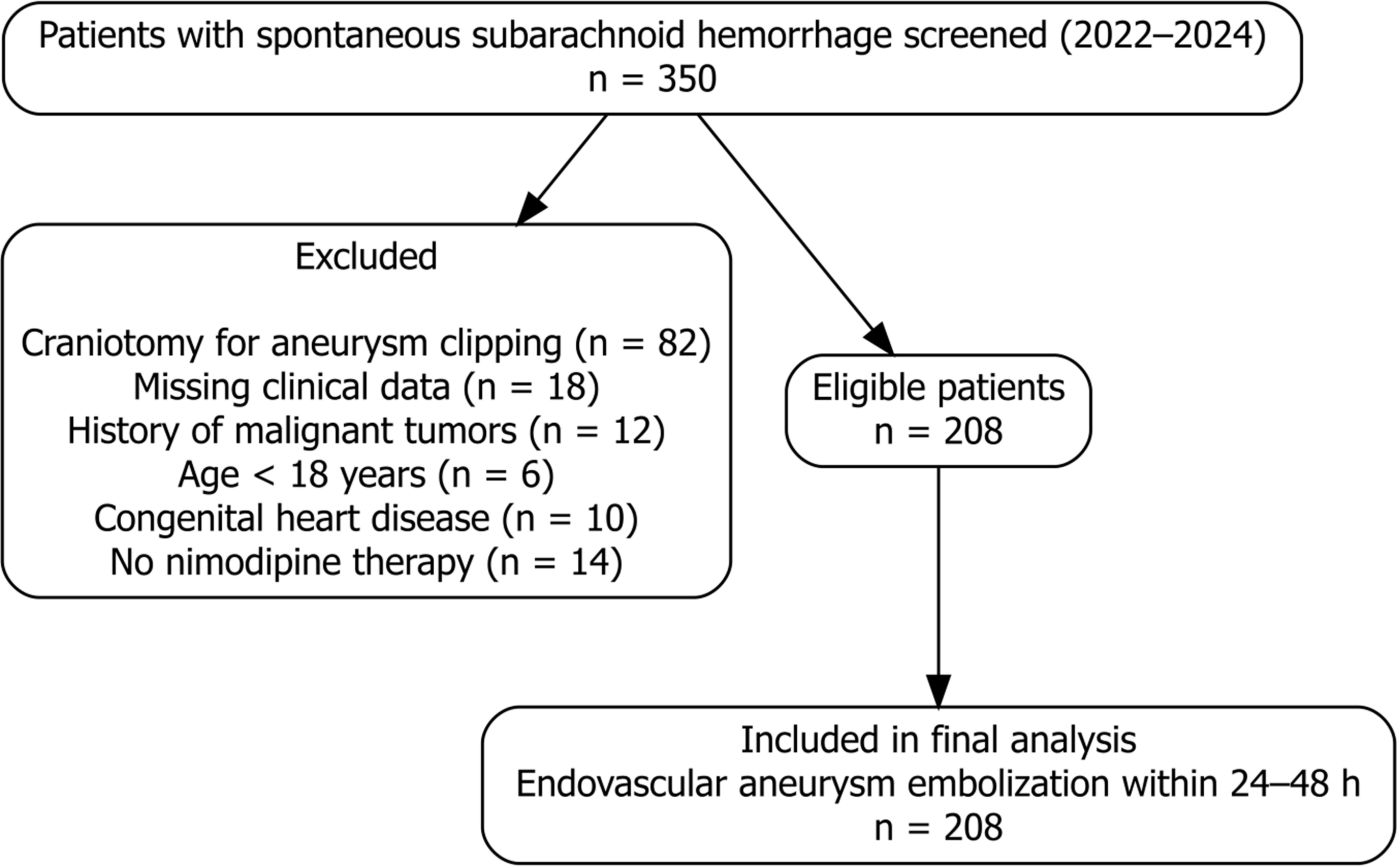
Flowchart of patient inclusion and exclusion. Patients with spontaneous subarachnoid hemorrhage screened between 2022 and 2024 were evaluated according to the predefined inclusion and exclusion criteria, including exclusion of pregnant patients if present. After applying the exclusion criteria, 208 patients who underwent endovascular aneurysm embolization within 24–48 h were included in the final analysis.

This single-center retrospective cohort study was approved by the local institutional ethics review board. Consecutive patients with aneurysmal subarachnoid hemorrhage (aSAH) admitted between 2022 and 2024 were screened. Eligible patients underwent endovascular aneurysm embolization within 24–48 h after initial presentation. After applying the predefined inclusion and exclusion criteria, 208 patients were included in the final analysis (see Figure 1).

Inclusion criteria included: (1) age at least 18 years; (2) confirmed spontaneous subarachnoid hemorrhage (SAH) by imaging tests; (3) endovascular embolization performed within 24–48 h of initial presentation; (4) sufficient clinical data and laboratory reports available; and (5) absence of prior intracranial surgery.

Exclusion criteria included: (1) open surgical treatment for aSAH, including craniotomy and aneurysm clipping; (2) substantial missing clinical or laboratory data; (3) history of malignant neoplasm; (4) congenital heart disease; (5) absence of prophylactic nimodipine treatment for cerebral vasospasm prevention; and (6) pregnancy.

### Collection of data

On the subject of collecting data on demographics and clinical variables, age, sex, smoking, alcohol intake history, hypertension, diabetes mellitus, and coronary artery disease were all variables collected as well as vital signs at the time of admission to the hospital (body temperature, heart rate, and blood pressure).

Radiologic variables included right optic nerve sheath diameter (RONSD), measured on admission computed tomography (CT) images, and hemorrhage severity assessed using the modified Fisher grading scale. RONSD measurements were performed independently by two physicians experienced in neurocritical-care imaging who were blinded to clinical outcomes; disagreements were resolved by consensus review. Neurological severity was assessed using the Hunt-Hess grading system and Glasgow Coma Scale (GCS) score.

In terms of clinical complications, the following were recorded: hydrocephalus, seizures, neurogenic pulmonary edema (NPE), and myocardial injury.

Laboratory parameters were obtained from the first blood sample drawn after intensive care unit admission. The selected variables reflected routinely available domains relevant to SAH-related systemic injury, including inflammation or infection (white blood cell count [WBC], neutrophil percentage [NEUT], PCT, and hsCRP), oxygen delivery and coagulation status (hemoglobin [HGB], platelet count [PLT], and D-dimer), tissue perfusion and electrolyte balance (lactate and sodium), renal function (creatinine), and myocardial injury (hs-cTnT). Calcium, potassium, and urea were not included in the predefined model because they were not part of the prespecified analysis set and were less consistently available across the cohort.

### Definition of myocardial injury

Myocardial injury was evaluated using the first hs-cTnT level measured after ICU admission. Because electrocardiographic and echocardiographic data were not consistently available in this retrospective cohort, myocardial injury was defined using a biomarker-based approach.

An hs-cTnT level ≥0.2 ng/mL (200 ng/L), approximately four times the upper limit of normal in our institutional laboratory, was considered indicative of myocardial injury. This definition reflects biomarker elevation suggestive of myocardial injury rather than a formal diagnosis of neurogenic stunned myocardium.

### Outcome definition

Study outcomes included myocardial injury and short-term neurological prognosis. Neurological outcome was assessed using the Glasgow Outcome Scale (GOS). GOS scores of 4–5 were classified as good prognosis, whereas GOS scores of 1–3 were classified as poor prognosis.

For regression analyses, myocardial injury was coded as 1 and absence of myocardial injury as 0. Poor prognosis (GOS 1–3) was coded as 1, and good prognosis (GOS 4–5) was coded as 0.

### Statistical analysis

All statistical analyses were conducted using R software version 4.4.1. Continuous variables were assessed for normality with the Shapiro–Wilk test. Normally distributed variables were planned to be summarized as mean +/− standard deviation, whereas non-normally distributed variables were summarized as median with interquartile range. Because all continuous variables presented in Tables 1, 2 showed non-normal distributions, they are reported as medians with 25th and 75th percentiles. Categorical variables are summarized as frequencies and percentages. Between-group comparisons were performed using the Mann–Whitney U test for non-normally distributed continuous variables and the chi-square test or Fisher’s exact test for categorical variables, as appropriate.

**Table 1:** Baseline clinical characteristics, imaging findings, and laboratory indicators according to myocardial injury status.

Variables	No myocardial injury (*n* = 159)	Myocardial injury (*n* = 49)	*p*
Sex: female, *n* (%)	104 (65.4)	35 (71.4)	0.543
Hypertension, *n* (%)	109 (68.6)	36 (73.5)	0.633
Diabetes mellitus, *n* (%)	19 (11.9)	7 (14.3)	0.853
Smoking history, *n* (%)	19 (11.9)	5 (10.2)	0.937
Alcohol intake history, *n* (%)	22 (13.8)	2 (4.1)	0.107
Coronary artery disease, *n* (%)	11 (6.9)	3 (6.1)	1
Neurogenic pulmonary edema, *n* (%)	2 (1.3)	8 (16.3)	<0.001
Seizure/epilepsy, *n* (%)	4 (2.5)	3 (6.1)	0.441
Hydrocephalus, *n* (%)	24 (15.1)	18 (36.7)	0.002
Aneurysm location category, *n* (%)	71 (44.7)	28 (57.1)	0.172
High modified Fisher grade, *n* (%)	45 (28.3)	26 (53.1)	0.002
High Hunt-Hess grade, *n* (%)	91 (57.2)	37 (75.5)	0.033
Age (years)	66.00 [57.00, 73.50]	71.00 [61.00, 78.00]	0.032
RONSD (mm)	5.96 [5.58, 6.57]	6.78 [6.09, 7.30]	<0.001
LONSD (mm)	6.10 [5.66, 6.83]	6.73 [6.14, 7.21]	0.001
GCS score	13.00 [11.00, 14.00]	9.00 [6.00, 13.00]	<0.001
Body temperature (deg C)	36.60 [36.50, 36.80]	36.70 [36.50, 36.90]	0.404
Heart rate (beats/min)	80.00 [72.50, 81.50]	80.00 [76.00, 88.00]	0.19
Systolic blood pressure (mmHg)	153.00 [140.00, 170]	167.00 [135.00, 185]	0.17
Diastolic blood pressure (mmHg)	88.00 [80.00, 100.00]	100.00 [80.00, 105.]	0.231
WBC (x10^9/L)	10.71 [7.98, 13.03]	12.38 [9.90, 17.18]	0.002
Neutrophils (%)	86.10 [80.75, 90.25]	89.00 [84.90, 91.40]	0.011
Hemoglobin (g/L)	120.0 [109.0, 132.0]	113.0 [106.0, 131.0]	0.31
Platelet count (x10^9/L)	197.0 [153.0, 242.0]	181.0 [138.0, 217.0]	0.128
Lactate (mmol/L)	1.30 [0.90, 2.35]	2.30 [1.40, 3.60]	<0.001
Sodium (mmol/L)	141.0 [139.0, 143.0]	141.0 [138.6, 144.0]	0.897
Creatinine (umol/L)	52.00 [44.00, 64.00]	58.00 [48.00, 73.00]	0.023
PCT (ng/mL)	0.08 [0.03, 0.21]	0.50 [0.19, 1.83]	<0.001
hsCRP (mg/L)	8.83 [3.22, 24.35]	44.30 [12.00, 85.40]	<0.001
D-dimer (ng/mL)	404 [230, 678]	1,951 [684, 3,378]	<0.001

Categorical variables are presented as *n* (%). Continuous variables were non-normally distributed and are presented as median [Q1, Q3]. Between-group comparisons used Mann–Whitney U tests for continuous variables and chi-square or Fisher’s exact tests for categorical variables. Abbreviations: CAD, coronary artery disease; NPE, neurogenic pulmonary edema; RONSD/LONSD, right/left optic nerve sheath diameter; GCS, Glasgow Coma Scale; WBC, white blood cell count; PCT, procalcitonin; hsCRP, high-sensitivity C-reactive protein; hs-cTnT, high-sensitivity cardiac troponin T. Aneurysm location and high-grade Fisher/Hunt-Hess variables were coded according to the prespecified data-extraction categories.

**Table 2:** Baseline clinical characteristics, imaging findings, and laboratory indicators according to short-term neurological prognosis at discharge.

Variables	Poor prognosis (GOS 1–3) (*n* = 78)	Good prognosis (GOS 4–5) (*n* = 130)	*p*
Sex: female, *n* (%)	52 (66.7)	87 (66.9)	1
Hypertension, *n* (%)	58 (74.4)	87 (66.9)	0.33
Diabetes mellitus, *n* (%)	15 (19.2)	11 (8.5)	0.04
Smoking history, *n* (%)	13 (16.7)	11 (8.5)	0.117
Alcohol intake history, *n* (%)	9 (11.5)	15 (11.5)	1
Coronary artery disease, *n* (%)	7 (9.0)	7 (5.4)	0.475
Neurogenic pulmonary edema, *n* (%)	6 (7.7)	2 (1.5)	0.063
Seizure/epilepsy, *n* (%)	2 (2.6)	3 (2.3)	1
Hydrocephalus, *n* (%)	37 (47.4)	5 (3.8)	<0.001
Aneurysm location category, *n* (%)	43 (55.1)	56 (43.1)	0.123
High modified Fisher grade, *n* (%)	43 (55.1)	28 (21.5)	<0.001
High Hunt-Hess grade, *n* (%)	69 (88.5)	59 (45.4)	<0.001
Age (years)	62.50 [53.00, 72.00]	71.50 [62.00, 77.75]	<0.001
RONSD (mm)	5.85 [5.52, 6.36]	6.70 [6.22, 7.15]	<0.001
LONSD (mm)	5.96 [5.65, 6.55]	6.85 [6.17, 7.03]	<0.001
GCS score	13.00 [12.00, 14.00]	9.00 [6.00, 11.00]	<0.001
Body temperature (deg C)	36.60 [36.50, 36.80]	36.65 [36.50, 36.90]	0.548
Heart rate (beats/min)	80.00 [71.00, 82.00]	80.00 [75.25, 86.00]	0.509
Systolic blood pressure (mmHg)	150.0 [135.0, 170.0]	160.0 [150.0, 182.25]	0.005
Diastolic blood pressure (mmHg)	89.00 [80.00, 100.75]	90.00 [80.00, 100.00]	0.819
WBC (x10^9/L)	10.68 [7.98, 12.87]	11.52 [9.16, 16.27]	0.009
Neutrophils (%)	85.90 [80.50, 89.97]	88.00 [84.45, 91.10]	0.019
Hemoglobin (g/L)	120.5 [110.0, 131.75]	115.5 [106.0, 130.75]	0.268
Platelet count (x10^9/L)	204.5 [158.25, 252.5]	177.0 [145.0, 223.0]	0.007
Lactate (mmol/L)	1.30 [0.90, 2.28]	2.10 [1.20, 3.53]	<0.001
Sodium (mmol/L)	141.0 [139.0, 143.0]	141.0 [139.0, 143.0]	0.793
hs-cTnT (ng/mL)	0.01 [0.00, 0.02]	0.05 [0.01, 0.75]	<0.001
Creatinine (umol/L)	50.50 [44.00, 60.75]	57.00 [48.25, 73.75]	0.002
PCT (ng/mL)	0.07 [0.01, 0.12]	0.37 [0.19, 1.03]	<0.001
hsCRP (mg/L)	7.39 [3.19, 11.97]	44.95 [26.90, 85.00]	<0.001
D-dimer (ng/mL)	367.5 [206.5, 578.75]	1,102 [594.8, 2996.7]	<0.001

Poor prognosis was defined as GOS 1-3 and good prognosis as GOS 4-5. Categorical variables are presented as *n* (%). Continuous variables were non-normally distributed and are presented as median [Q1, Q3]. Between-group comparisons used Mann–Whitney U tests for continuous variables and chi-square or Fisher’s exact tests for categorical variables. Abbreviations: CAD, coronary artery disease; NPE, neurogenic pulmonary edema; RONSD/LONSD, right/left optic nerve sheath diameter; GCS, Glasgow Coma Scale; WBC, white blood cell count; PCT, procalcitonin; hsCRP, high-sensitivity C-reactive protein; hs-cTnT, high-sensitivity cardiac troponin T. Aneurysm location and high-grade Fisher/Hunt-Hess variables were coded according to the prespecified data-extraction categories.

Univariable logistic regression was first performed to identify variables potentially associated with myocardial injury and short-term prognosis. A two-sided *p* value <0.05 was considered statistically significant throughout the study. A univariable *p* value <0.10 was used only as a liberal screening criterion for candidate variable selection and not as a threshold for statistical significance. Clinically important variables, including age and sex, were considered during model development regardless of their univariable *p* values. Because of the limited number of outcome events, parsimonious final models were developed to reduce overfitting risk and improve model stability.

Multicollinearity among variables included in the final models was evaluated using variance inflation factors (VIFs). A VIF value <5 was considered to indicate no relevant multicollinearity. The events-per-variable (EPV) ratio was considered during model construction, and final models were kept parsimonious because the number of myocardial injury events was limited. The Box-Tidwell linearity assumption for continuous predictors was not formally evaluated for every candidate predictor and is therefore acknowledged as a methodological limitation.

Because some binary predictors had few outcome events, Firth penalized logistic regression was additionally applied as a sensitivity analysis for the two binary outcomes: myocardial injury and poor short-term prognosis. The sensitivity models used the same covariates as the primary multivariable models. Since hsCRP showed a right-skewed distribution, it was entered into the regression models as a per 10-unit increase.

Nomograms were generated from the final multivariable logistic regression models as exploratory tools for early risk stratification and clinical decision support research. Model discrimination was assessed using receiver operating characteristic (ROC) curves and the area under the curve (AUC). Calibration was examined by internal bootstrap resampling with 1,000 repetitions. Decision curve analysis (DCA) was used to estimate potential clinical net benefit. Because no independent external cohort was available, these models should be interpreted as preliminary and require external validation before clinical implementation.

All statistical tests were two-sided, and *p* < 0.05 was considered statistically significant.

### Clinical management

All patients received standardized treatment according to institutional protocols, including hemodynamic monitoring, blood pressure management, nimodipine administration for cerebral vasospasm prophylaxis, fluid and electrolyte management, and supportive intensive care treatment when necessary. Complications such as hydrocephalus, epilepsy, and neurogenic pulmonary edema were treated according to the patients’ clinical conditions.

## Results

A total of 208 patients with aneurysmal subarachnoid hemorrhage were included in the present study. Among them, 49 patients (23.6%) developed myocardial injury, whereas 159 patients (76.4%) did not. In addition, 130 patients (62.5%) had a good prognosis and 78 patients (37.5%) had a poor prognosis.

The baseline characteristics of patients with and without myocardial injury are summarized in Table 1. Univariable and multivariable logistic regression analyses for myocardial injury are presented in Table 3. The baseline characteristics of patients stratified by short-term prognosis are shown in Table 2, and the corresponding univariable and multivariable logistic regression analyses are presented in Table 4.

**Table 3:** Univariable and multivariable logistic regression analyses for myocardial injury in patients with aSAH.

Variables	Univariable		Multivariable	
	OR (95% CI)	*p*	OR (95% CI)	*p*
Sex: female	1.32 (0.66–2.66)	0.435		
Hypertension	1.27 (0.62–2.60)	0.513		
Diabetes mellitus	1.23 (0.48–3.12)	0.666		
Smoking history	0.84 (0.30–2.37)	0.738		
Alcohol intake history	0.26 (0.06–1.17)	0.080		
Coronary artery disease	0.88 (0.23–3.28)	0.846		
Neurogenic pulmonary edema	15.32 (3.13–74.90)	<0.001	18.87 (3.18–112.03)	0.001
Seizure/epilepsy	2.53 (0.55–11.70)	0.236		
Hydrocephalus	3.27 (1.58–6.74)	0.001	1.13 (0.39–3.26)	0.820
Aneurysm location category	1.65 (0.87–3.15)	0.128		
High modified Fisher grade	2.86 (1.48–5.53)	0.002	0.46 (0.14–1.53)	0.206
High Hunt-Hess grade	2.30 (1.12–4.75)	0.024	0.29 (0.08–1.02)	0.054
Age	1.03 (1.00–1.05)	0.061		
RONSD	3.20 (1.96–5.23)	<0.001	4.20 (1.58–11.19)	0.004
LONSD	2.27 (1.44–3.57)	<0.001	0.54 (0.21–1.36)	0.189
GCS	0.76 (0.69–0.85)	<0.001	0.84 (0.67–1.04)	0.110
Body temperature	1.53 (0.67–3.49)	0.316		
Heart rate	1.02 (0.99–1.04)	0.218		
Systolic blood pressure	1.01 (1.00–1.02)	0.179		
Diastolic blood pressure	1.01 (0.99–1.03)	0.372		
WBC	1.00 (0.99–1.01)	0.764		
Neutrophils	1.04 (0.99–1.08)	0.093		
Hemoglobin	1.00 (0.98–1.01)	0.758		
Platelet count	1.00 (0.99–1.00)	0.108		
Lactate	1.43 (1.18–1.72)	<0.001	1.20 (0.93–1.55)	0.157
Sodium	1.02 (0.92–1.12)	0.751		
Creatinine	1.01 (1.00–1.02)	0.030	1.00 (1.00–1.01)	0.348
PCT	2.14 (1.42–3.21)	<0.001	1.45 (1.01–2.08)	0.045
hsCRP	1.02 (1.01–1.03)	<0.001	1.01 (1.00–1.02)	0.274
D-dimer	1.001 (1.000–1.001)	<0.001	1.000 (1.000–1.001)	0.077

ORs are reported with 95% CIs, and *p* values are reported without the “P =” prefix. OR, odds ratio; CI, confidence interval. *p* values are shown without the ‘P =” prefix. Variables were considered for multivariable analysis based on clinical relevance, prior evidence, or liberal univariable screening (*p* < 0.10), while *p* < 0.05 was used as the threshold for statistical significance. Age and sex were considered during model development regardless of univariable significance. Aneurysm location and high-grade modified Fisher/Hunt-Hess variables were coded according to the prespecified data-extraction categories.

**Table 4:** Univariable and multivariable logistic regression analyses for short-term neurological prognosis.

Variables	Univariable		Multivariable	
	OR (95% CI)	*p*	OR (95% CI)	*p*
Sex: female	1.01 (0.56–1.84)	0.970		
Hypertension	0.70 (0.37–1.30)	0.260		
Diabetes mellitus	0.39 (0.17–0.90)	0.026	0.73 (0.21–2.50)	0.615
Smoking history	0.46 (0.20–1.09)	0.078		
Alcohol intake history	1.00 (0.42–2.41)	1.00		
Coronary artery disease	0.58 (0.19–1.71)	0.322		
Neurogenic pulmonary edema	0.19 (0.04–0.95)	0.044	0.67 (0.03–14.24)	0.796
Seizure/epilepsy	0.90 (0.15–5.49)	0.907		
Hydrocephalus	0.04 (0.02–0.12)	<0.001	0.06 (0.01–0.25)	<0.001
Aneurysm location category	0.62 (0.35–1.08)	0.093		
High modified Fisher grade	0.22 (0.12–0.41)	<0.001	1.74 (0.54–5.64)	0.356
High Hunt-Hess grade	0.11 (0.05–0.24)	<0.001	2.07 (0.45–9.54)	0.353
Age	0.94 (0.92–0.97)	<0.001	0.94 (0.90–0.99)	0.011
RONSD	0.28 (0.18–0.44)	<0.001	0.54 (0.16–1.82)	0.321
LONSD	0.32 (0.21–0.50)	<0.001	1.33 (0.37–4.74)	0.661
GCS	1.74 (1.50–2.03)	<0.001	1.76 (1.27–2.43)	<0.001
Body temperature	0.83 (0.39–1.77)	0.628		
Heart rate	0.98 (0.96–1.00)	0.110		
Systolic blood pressure	0.98 (0.97–1.00)	0.006	0.99 (0.97–1.02)	0.0582
Diastolic blood pressure	1.00 (0.98–1.02)	0.819		
WBC	1.00 (1.00–1.01)	0.612		
Neutrophils	0.98 (0.95–1.01)	0.190		
Hemoglobin	1.00 (0.99–1.01)	0.937		
Platelet count	1.01 (1.00–1.01)	0.014	1.00 (1.00–1.01)	0.374
Lactate	0.72 (0.59–0.87)	<0.001	1.01 (0.74–1.40)	0.930
Sodium	0.98 (0.90–1.06)	0.574		
hs-cTnT	0.53 (0.37–0.76)	<0.001	1.11 (0.84–1.48)	0.455
Creatinine	0.98 (0.97–1.00)	0.010	1.00 (0.99–1.02)	0.885
PCT	0.29 (0.15–0.56)	<0.001	1.42 (0.88–2.31)	0.155
hsCRP per 10 mg/L	1.67 (1.42–1.96)	<0.001	1.51 (1.24–1.86)	<0.001
D-dimer	1.001 (1.000–1.001)	<0.001	1.000 (0.999–1.001)	0.663

High hsCRP, lower GCS score, older age, and hydrocephalus were associated with poorer short-term prognosis. OR, odds ratio; CI, confidence interval. *p* values are shown without the “P =” prefix. Variables were considered for multivariable analysis based on clinical relevance, prior evidence, or liberal univariable screening (*p* < 0.10), while *p* < 0.05 was used as the threshold for statistical significance. Age and sex were considered during model development regardless of univariable significance. Aneurysm location and high-grade modified Fisher/Hunt-Hess variables were coded according to the prespecified data-extraction categories.

To reduce potential small-sample bias and quasi-separation caused by sparse binary events, Firth penalized logistic regression was additionally performed as a sensitivity analysis using the same covariates included in the primary multivariable logistic regression models. The results remained generally consistent with the primary analyses (Table 5). Neurogenic pulmonary edema (NPE), procalcitonin (PCT), and right optic nerve sheath diameter (RONSD) remained independently associated with myocardial injury. In the prognostic model, hydrocephalus, hsCRP, age, and Glasgow Coma Scale (GCS) score also remained significantly associated with short-term prognosis.

**Table 5:** Firth penalized logistic regression sensitivity analyses for the two binary outcomes: myocardial injury and poor short-term prognosis.

Outcome	Variable	Beta firth	OR firth	Lower 95 CI firth	Upper 95 CI firth	*p* value firth
Myocardial injury	NPE (Yes)	2.675	14.515	3.241	94.574	<0.001
Myocardial injury	PCT	0.516	1.675	1.238	2.483	0.001
Myocardial injury	RONSD	1.003	2.730	1.663	4.624	<0.001
Poor prognosis	Hydrocephalus (Yes)	2.373	10.734	3.447	38.611	<0.001
Poor prognosis	hsCRP_10	0.349	1.417	1.221	1.698	<0.001
Poor prognosis	GCS	−0.440	0.644	0.5261	0.771	<0.001
Poor prognosis	Age	0.050	1.052	1.015	1.099	0.004

Firth penalized logistic regression was used as a sensitivity analysis for both binary outcomes: myocardial injury and poor short-term prognosis. OR, odds ratio; CI, confidence interval; PCT, procalcitonin; RONSD, right optic nerve sheath diameter; hsCRP_10, high-sensitivity C-reactive protein per 10 mg/L; GCS, Glasgow Coma Scale.

Multicollinearity assessment demonstrated no evidence of significant collinearity among variables included in the final multivariable models, with all variance inflation factor (VIF) values close to 1 (Table 6).

**Table 6:** Variance inflation factor (VIF) analysis for variables retained in the final multivariable regression models.

Variable	VIF
Hydrocephalus	1.040
hsCRP_10	1.026
GCS	1.026
Age	1.030
NPE	1.015
PCT	1.009
RONSD	1.024

VIF, variance inflation factor. All VIF values were <5, indicating no relevant multicollinearity among variables retained in the final multivariable models.

**Figure 2:**
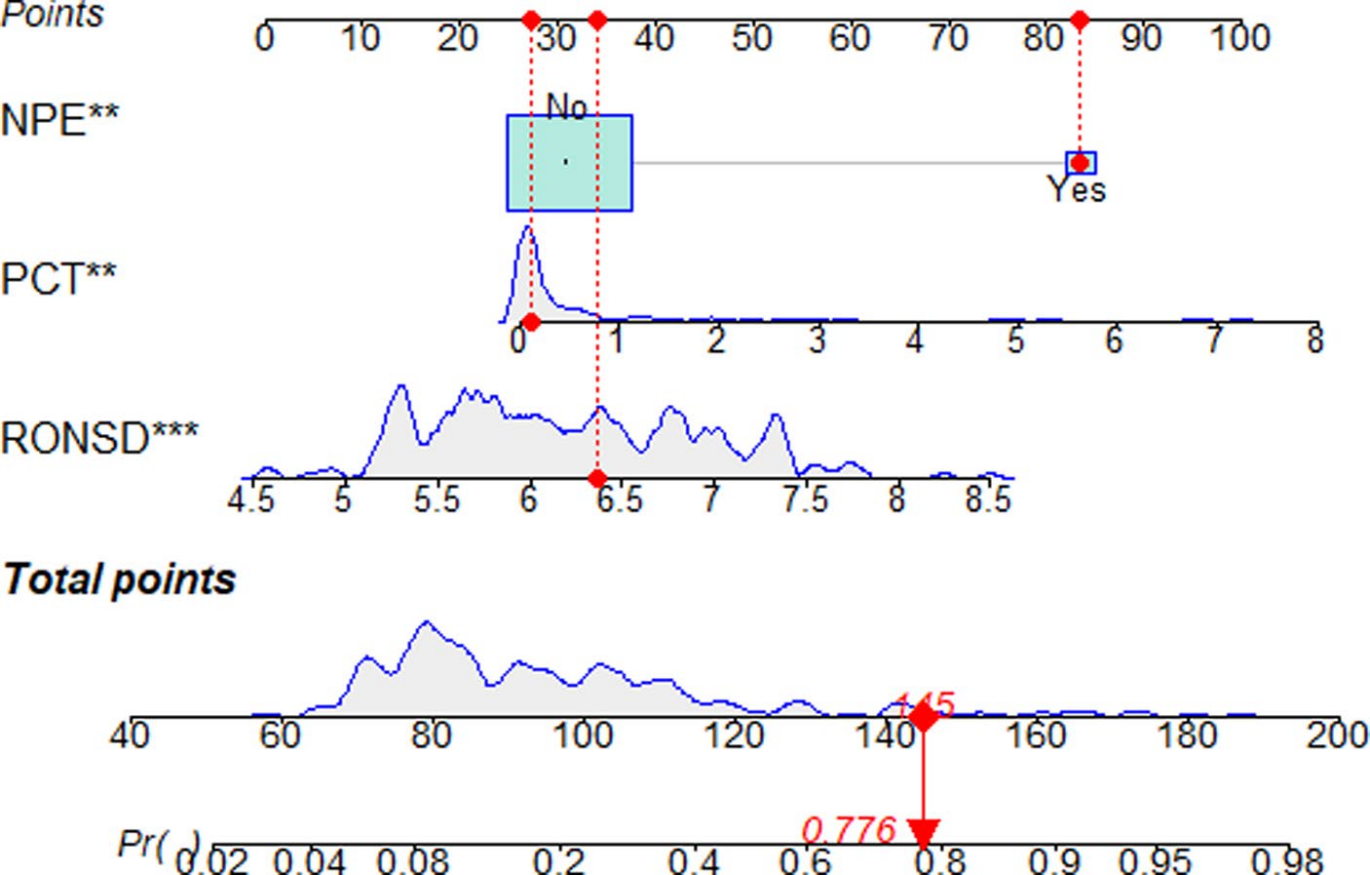
Exploratory nomogram for predicting short-term neurological prognosis at hospital discharge in patients with aSAH. The model incorporates age, hydrocephalus, GCS score, and hsCRP and requires external validation before routine clinical use.

**Figure 3:**
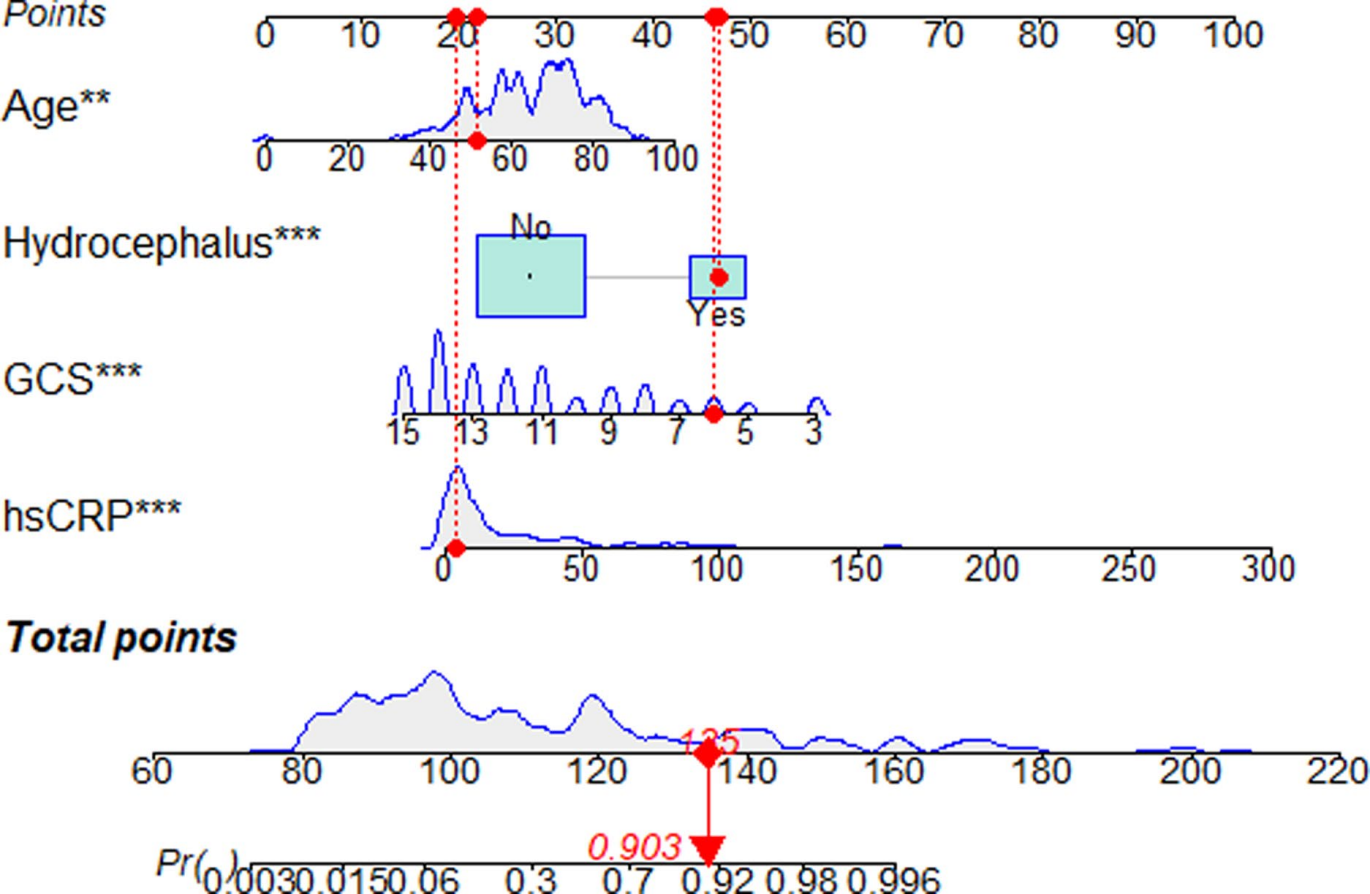
Exploratory nomogram for predicting myocardial injury risk in patients with aSAH. The model incorporates NPE, PCT, and RONSD and is intended for preliminary risk stratification pending external validation.

**Figure 4:**
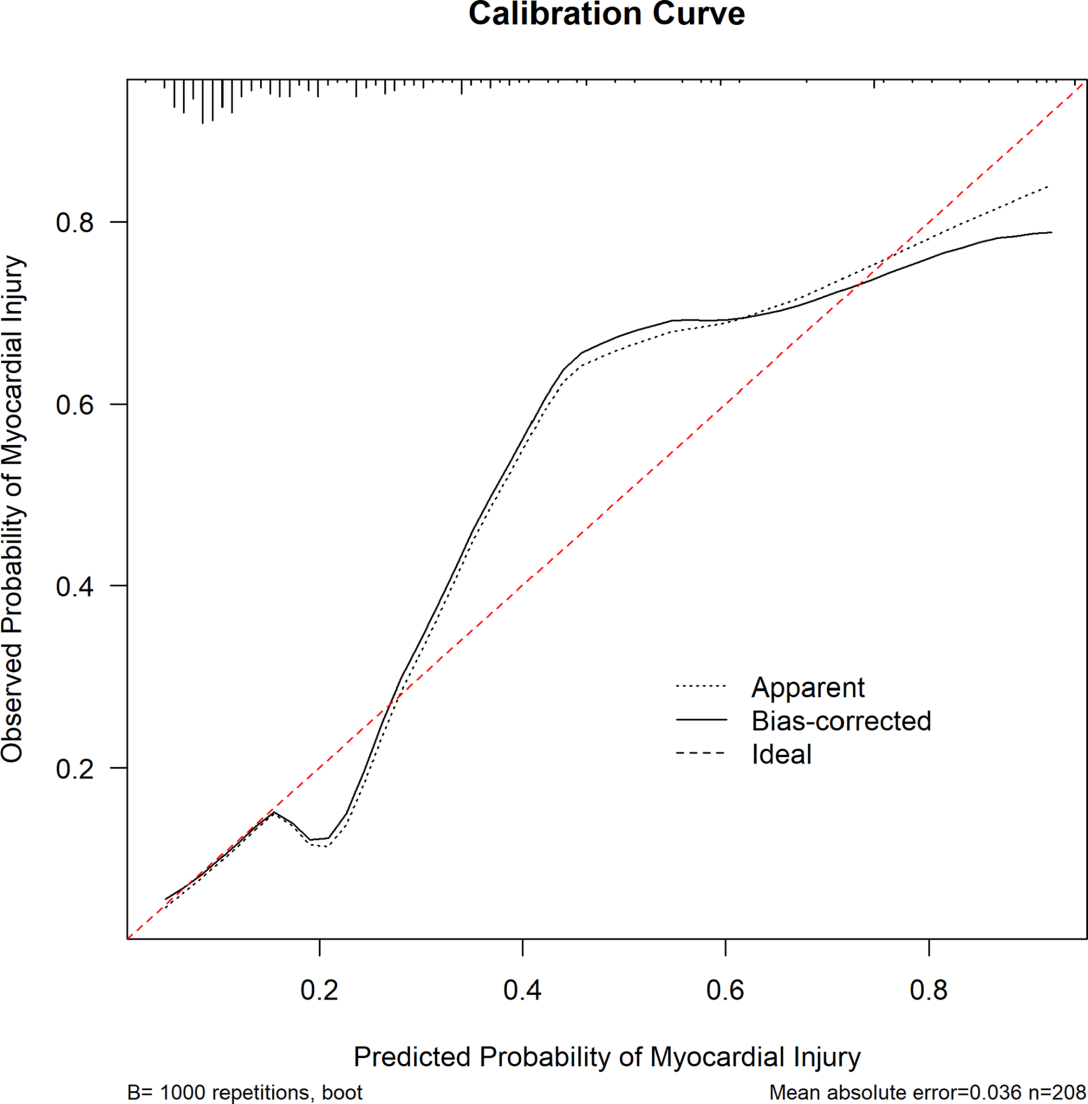
Calibration curve for myocardial injury risk prediction nomogram. Calibration curve assesses agreement of predicted/observed probabilities of myocardial injury in SAH patients diagnosed with myocardial injury according to nomogram. The solid line represents bias-corrected estimates; dotted line represents apparent performance; dashed diagonal line represents perfect calibration. Close alignment of solid curve with dashed diagonal indicates good calibration/reliable model. Mean absolute error = 0.036 indicates there is very little deviation between predicted and observed outcomes.

**Figure 5:**
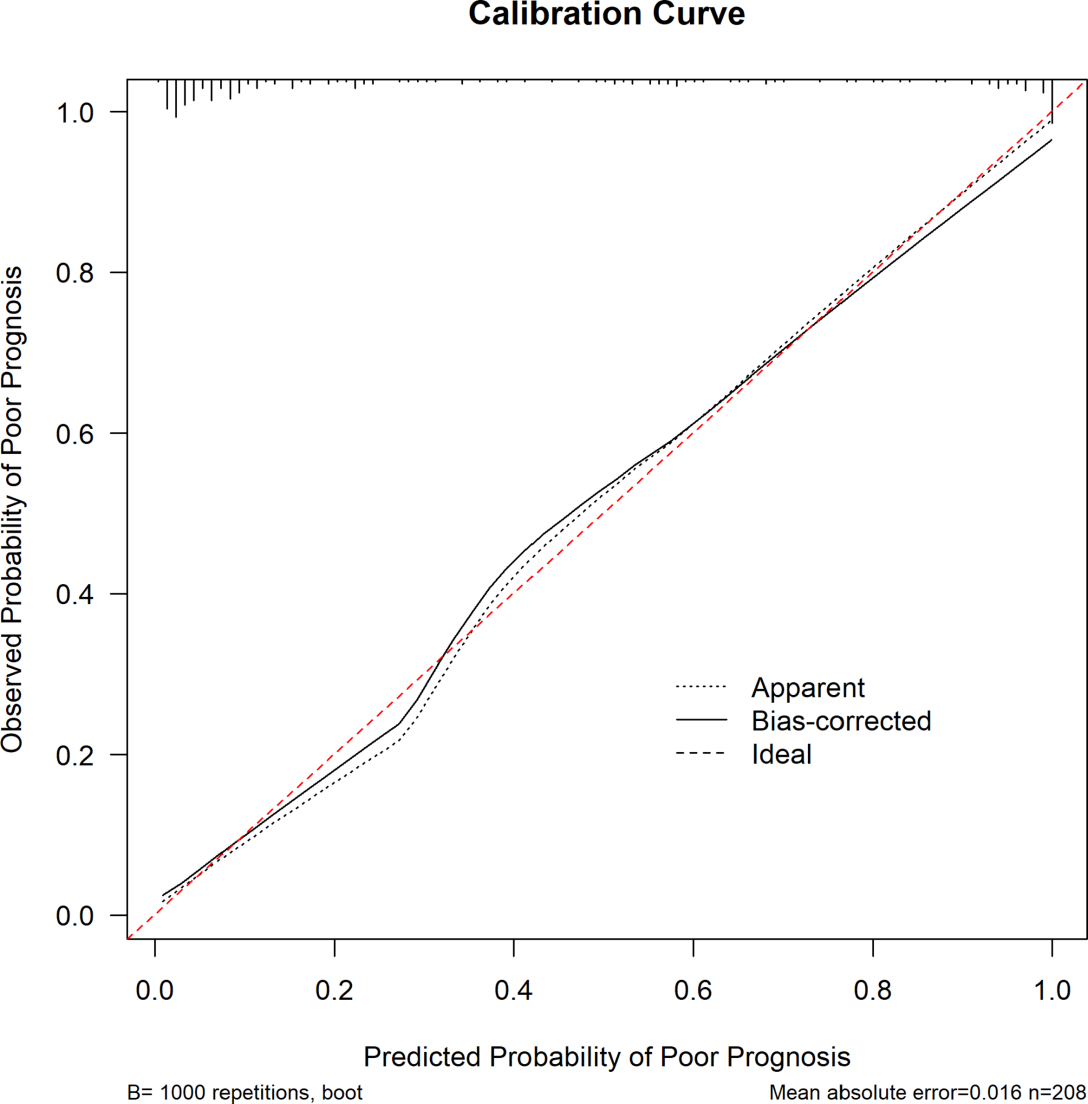
It depicts the calibration curve for predicting short-term prognosis based on the nomogram in patients with SAH. According to this graph, there appears to be good agreement between the predicted risk and the actual risk (measured outcomes) for this population. The bias-corrected curve closely approximated the ideal reference line, indicating excellent calibration and validity of the model. The mean absolute error was found to be 0.016 based on the bootstrap sample of the data (*n*) of 208 resamples obtained from the sample of 1,000.

**Figure 6:**
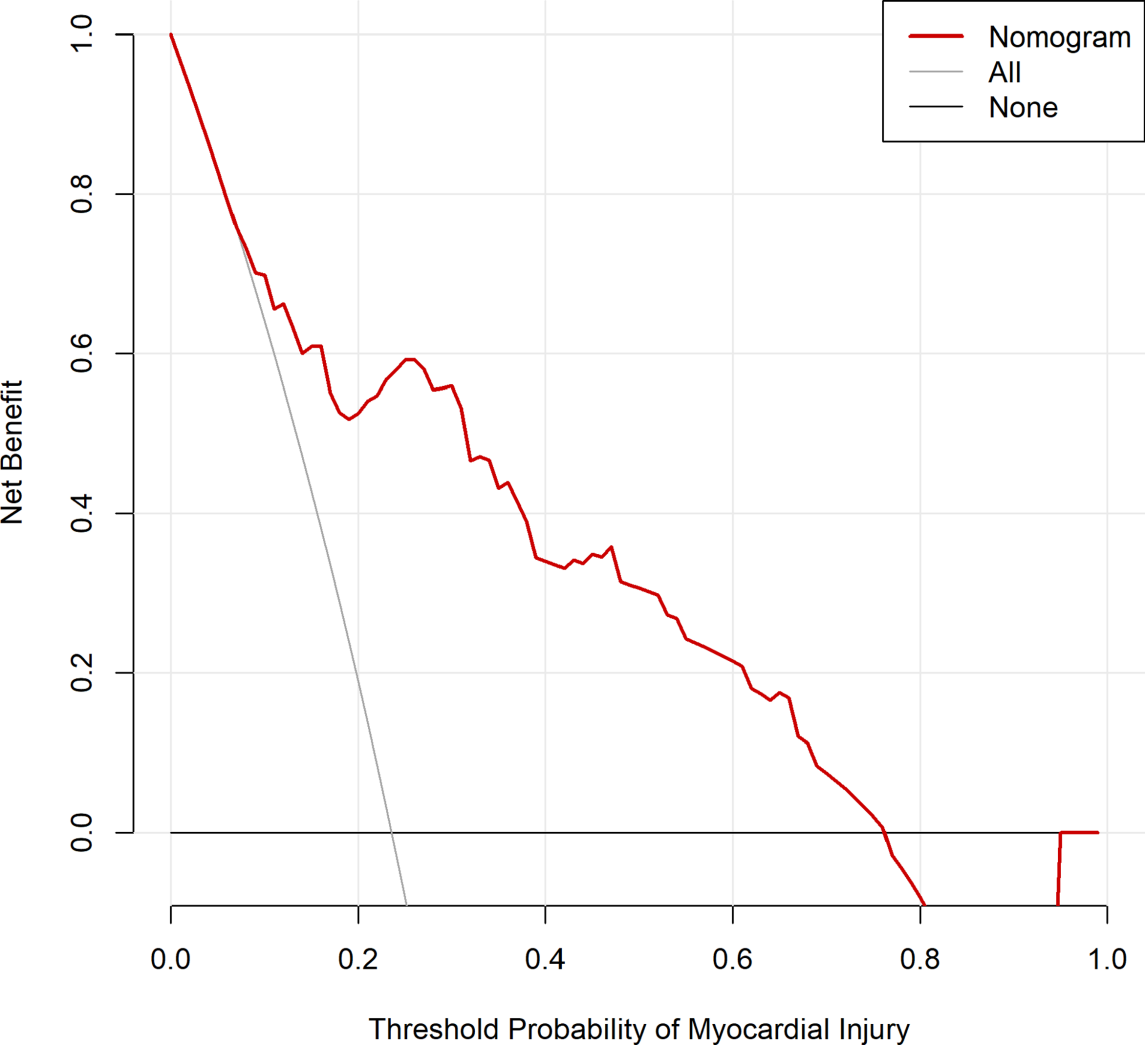
It depicts the decision curve analysis (DCA) performed on the myocardial injury risk prediction nomogram developed for patients with SAH. The DCA illustrates the standardized net benefit (SNB) of the myocardial injury risk prediction nomogram based on a variety of threshold probabilities (0–1) for use when predicting myocardial injury. The myocardial injury risk prediction nomogram demonstrated a net benefit greater than the treat-all and treat-none strategies at threshold probabilities approximately between 0.10 to 0.75. Therefore, these results suggest that the myocardial injury risk prediction nomogram has significant clinical utility in assisting healthcare providers in assessing risk and making decisions regarding patients with SAH.

**Figure 7:**
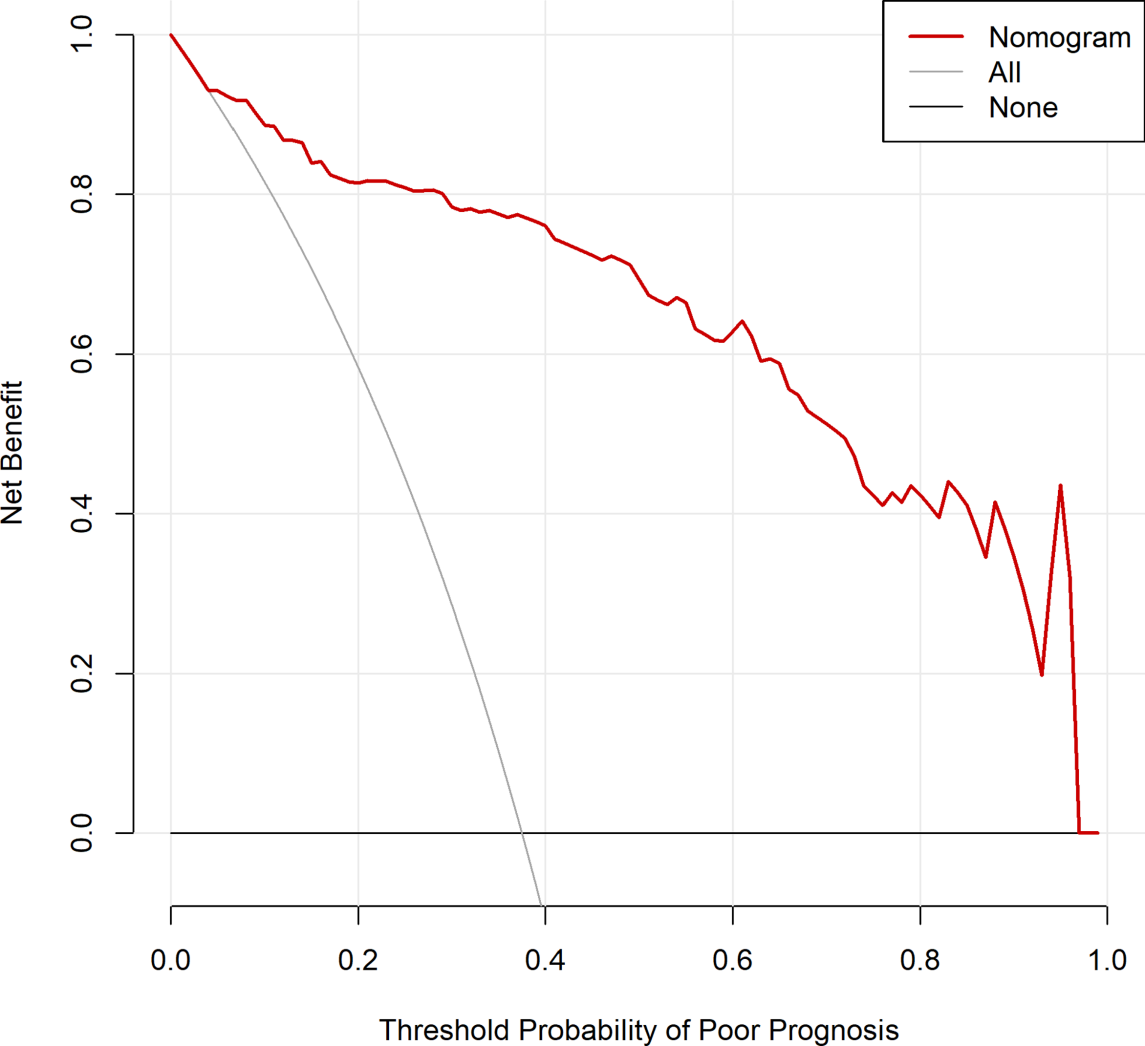
The short-term prognosis nomogram was used to assess decision curves (DCA) of primary outcome data in subarachnoid hemorrhage patients to identify potential clinical utility for guided systematic risk assessments and/or treatment selections. The short-term prognosis nomogram provided greater than expected “net” benefit relative to both treat-all and treat-none based treatment selection strategies over a wide range of threshold probabilities (from as low as 0.10 to as high as 0.90).

**Figure 8:**
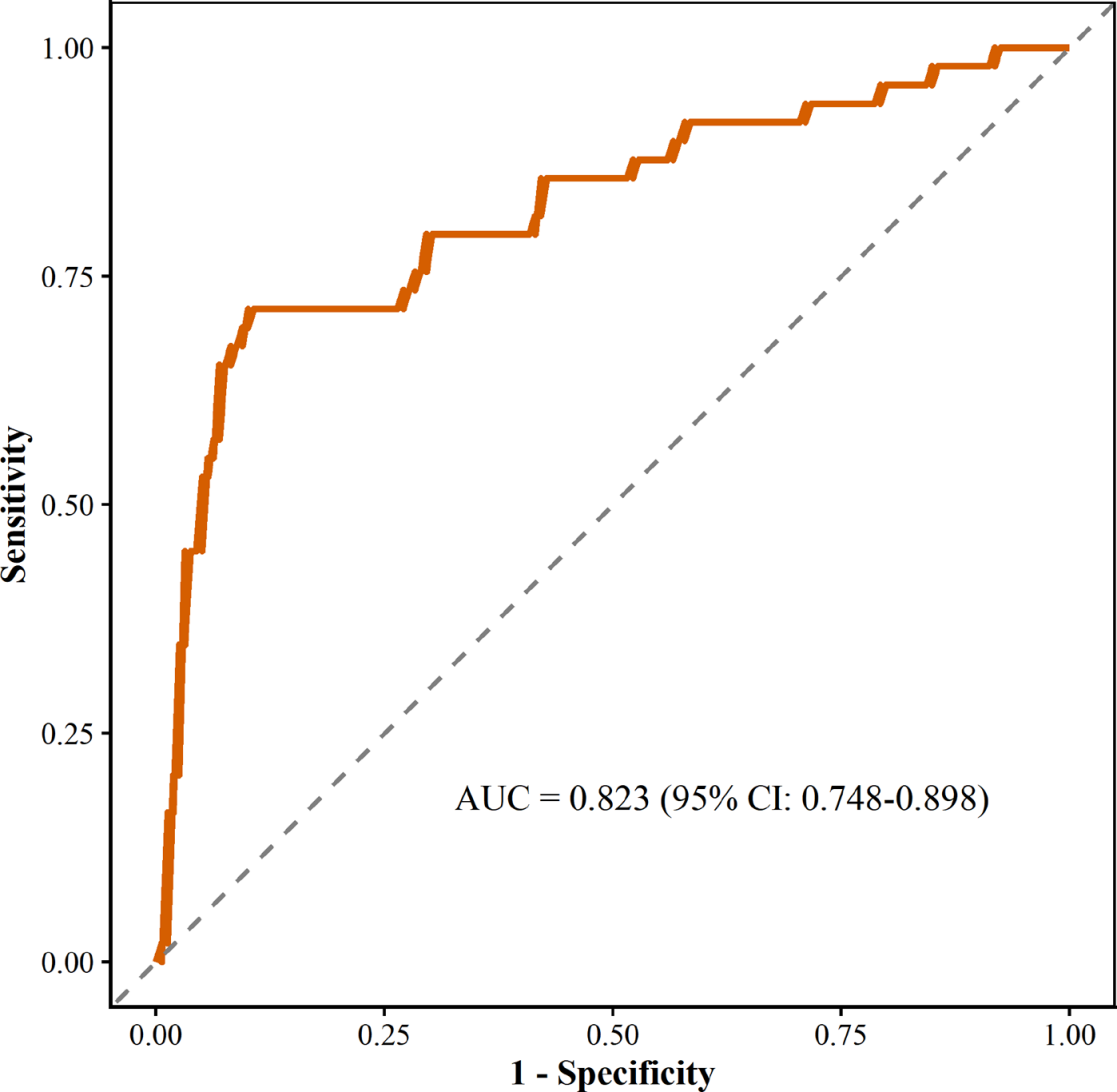
The myocardial injury prediction multivariable logistic regression model was found to possess good discrimination ability (AUC = 0.823, 95% CI = 0.748–0.898) as shown by the area under the receiver operating characteristic (ROC) curve.

**Figure 9:**
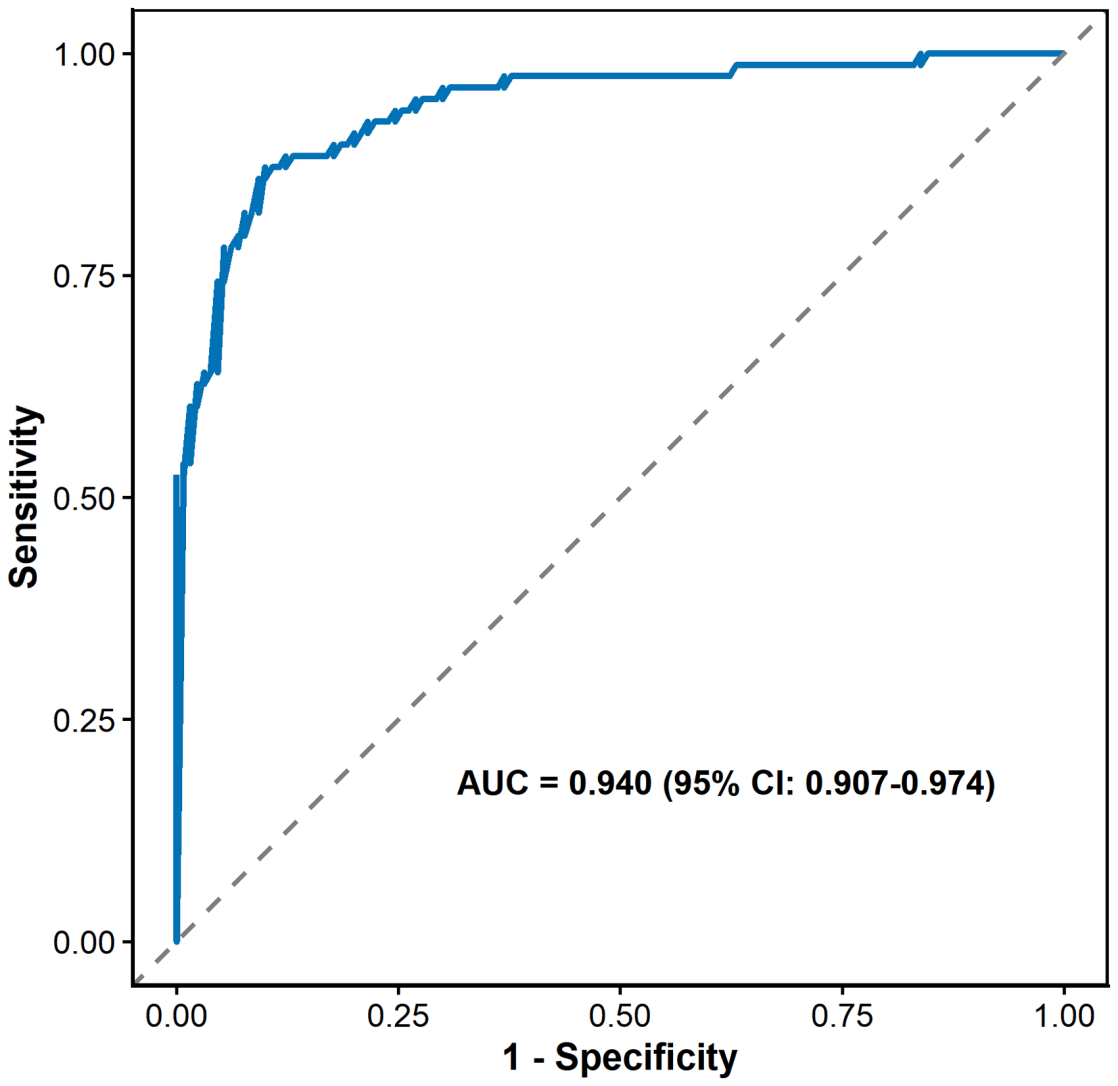
The poor prognosis prediction multivariable logistic regression model exhibited excellent discrimination ability (AUC = 0.940, 95% CI = 0.907–0.974) as presented by the area under the receiver operating characteristic (ROC) curve.

Nomograms were subsequently constructed based on the final multivariable logistic regression models for myocardial injury and short-term prognosis (Figures 2–9). In the myocardial injury model, NPE, PCT, and RONSD were identified as independent statistical predictors. In the prognostic model, hsCRP, GCS score, age, and hydrocephalus were identified as independent statistical predictors of poor short-term prognosis. After rescaling, hsCRP was entered into the model per 10-unit increase, and each 10-unit increase in hsCRP was associated with an increased risk of poor short-term prognosis (adjusted OR 1.51, 95% CI 1.24–1.86, *p* < 0.001). The nomograms are intended for hypothesis generation and early risk stratification research and require independent external validation before use as routine clinical decision tools.

Model discrimination was evaluated using receiver operating characteristic (ROC) curve analysis, while calibration performance was assessed using bootstrap resampling calibration curves. Decision curve analysis (DCA) was additionally performed to evaluate the potential clinical utility of the models; however, DCA findings should be regarded as preliminary because prospective impact testing and external validation were not performed.

## Discussion

Subarachnoid hemorrhage is a highly lethal and disabling disease. Poor outcomes are concentrated during hospitalization and the early post-discharge period, with reported early mortality of approximately 21% and intensive-care mortality of approximately 17% (22). Therefore, timely identification of factors associated with early deterioration is clinically important. In the present cohort, 49 of 208 patients (23.6%) developed biomarker-defined myocardial injury, which is broadly consistent with previous reports (17). Multivariable analysis showed that RONSD (OR = 4.20, 95% CI: 1.58–11.19, *p* = 0.004), PCT (OR = 1.45, 95% CI: 1.01–2.08, *p* = 0.045), and NPE (OR = 18.87, 95% CI: 3.18–112.03, *p* = 0.001) were independently associated with myocardial injury. These findings support a multi-parameter approach to identifying patients at increased risk, while recognizing that the observational design does not establish causality.

In contrast to studies focusing on a single index, the present analysis integrated imaging, inflammatory, and cardiopulmonary variables into the myocardial injury risk assessment model. RONSD at admission showed good predictive performance for myocardial injury, and the underlying explanation warrants further study. Optic nerve sheath diameter has been used as a noninvasive marker of intracranial pressure, and increased ONSD has been associated with worse outcomes in patients with subarachnoid hemorrhage (23, 24). We hypothesize that higher intracranial pressure may be associated with a more intense stress response and catecholamine release, leading to increased sympathetic activity and decreased parasympathetic activity, which may contribute to myocardial injury. At hospital discharge, hsCRP, GCS score, age, and hydrocephalus were associated with short-term prognosis. These findings support early multi-indicator monitoring in patients with SAH, but they should be interpreted as associations rather than direct causal effects.

Recent studies have further clarified potential mechanisms of neurogenic myocardial injury after SAH. Intense sympathetic activation and catecholamine surge are widely considered major contributors, while inflammatory responses, oxidative stress, and microcirculatory dysfunction may also be involved (25, 26). Epidemiological and mechanistic studies suggest that SAH prognosis is associated with local and systemic inflammation, delayed cerebral vasospasm, cardiomyocyte apoptosis, and calcium overload (12, 27). These findings are consistent with the observed association between hsCRP levels and prognosis in our cohort. PCT elevation may reflect infection, systemic inflammatory activation, or immune dysregulation, and may therefore represent a marker associated with neurogenic myocardial injury rather than a proven causal factor. Hydrocephalus, age, GCS score, and Hunt-Hess classification are established indicators of neurological injury severity and outcome (28, 29). Overall, multiparameter assessment combining neurological scores, biochemical markers, and imaging findings may improve early risk stratification and reduce reliance on single indicators.

Few studies have specifically evaluated short-term prognosis in patients with SAH complicated by myocardial injury, although early mortality is clinically important. Current management of SAH includes early aneurysm treatment, intracranial pressure control, vasospasm prevention, and comprehensive supportive care. In this study, only patients treated with endovascular embolization were included to reduce heterogeneity related to surgical modality. For myocardial injury, treatment remains primarily supportive. Emerging evidence has explored beta-blockers, antioxidants, milrinone, levosimendan, and anti-inflammatory strategies, but their roles require further confirmation (9, 30). Active management of poor prognostic factors, including hydrocephalus and low GCS score, remains critical; timely ventricular drainage, intracranial pressure stabilization, and correction of fluid and electrolyte abnormalities may support neurological recovery. In addition, analysis of the MIMIC database suggested that statin use may be associated with improved mortality outcomes in critically ill patients with SAH (22). Future management strategies should be tested prospectively before they are incorporated into routine practice.

Current research on SAH-associated myocardial injury is moving toward multi-indicator and multidimensional assessment. Cardiac complications after SAH encompass a spectrum of abnormalities, including biomarker-defined myocardial injury, cardiac dysfunction, and stress cardiomyopathy, and their diagnosis and management require the integration of cardiac biomarkers, electrocardiographic findings, and echocardiographic features (31). Previous studies have examined the relationships of cardiac biomarkers and cardiovascular dysfunction with neurogenic pulmonary edema, functional outcomes, and long-term prognosis after SAH (32–38). Takotsubo syndrome represents a distinct stress-related cardiac phenotype that may occur after SAH; however, its formal diagnosis requires syndrome-specific clinical and imaging criteria and should not be inferred from troponin elevation alone (39–41).

Advances in imaging, molecular biomarkers, and statistical modeling have encouraged the development of risk prediction models incorporating clinical variables, biomarkers, and physiological parameters. Markers of myocardial injury, echocardiographic findings, inflammatory indicators, and neurological indicators such as ONSD and NPE have gained attention as potential components of early risk stratification. However, the nomograms in this study were developed and internally validated in a single-center retrospective cohort. Therefore, they should be considered exploratory tools for early bedside risk stratification, clinical decision-support research, and future validation studies rather than instruments for immediate widespread clinical implementation.

### Research limitations

Several limitations of this study should be acknowledged. First, this was a single-center retrospective cohort study, which may be subject to selection and information bias and limits the generalizability of the findings to other hospitals, healthcare systems, imaging protocols, and perioperative management settings. Second, the sample size was relatively limited, particularly in the myocardial injury subgroup, and several variables exhibited wide confidence intervals, indicating statistical uncertainty and possible model instability. Third, although bootstrap internal validation and Firth penalized logistic regression were performed, internal validation cannot replace external validation in an independent population. The EPV ratio was limited relative to the number of candidate predictors considered during screening, and the findings should therefore be interpreted cautiously. Fourth, the Box-Tidwell linearity assumption for all continuous variables was not formally evaluated, which may influence the stability of logistic regression estimates when physiologically related variables are included. Fifth, myocardial injury was primarily evaluated using high-sensitivity troponin levels, whereas electrocardiographic and echocardiographic findings were not incorporated because these data were heterogeneous and incompletely available. Therefore, the findings mainly reflect biomarker-defined myocardial injury rather than a comprehensive syndrome-level diagnosis. Finally, this study focused only on short-term outcomes and lacked long-term follow-up data; therefore, the impact of myocardial injury on long-term neurological outcomes requires further investigation.

## Conclusion

In this single-center retrospective cohort, right optic nerve sheath diameter (RONSD), procalcitonin (PCT), and neurogenic pulmonary edema (NPE) were independently associated with myocardial injury in patients with aneurysmal subarachnoid hemorrhage (aSAH). High-sensitivity C-reactive protein (hsCRP), Glasgow Coma Scale (GCS) score, age, and hydrocephalus were associated with short-term neurological prognosis. Myocardial injury was not independently associated with short-term prognosis after adjustment, suggesting that it may reflect overall disease severity rather than independently determine early outcomes. Because the study was retrospective and observational, these findings should be interpreted as associations rather than causal relationships. The proposed nomograms remain preliminary and require external validation before routine clinical use.

### Future perspectives

Future research should prioritize multicenter prospective studies with larger sample sizes and independent external validation cohorts to assess the stability, calibration, and transportability of the proposed models. Prospective impact studies are also needed to determine whether nomogram-guided risk stratification improves clinical decision-making or patient outcomes. Advanced imaging techniques, such as cardiac magnetic resonance imaging (CMR), together with dynamic cardiac biomarkers and hemodynamic parameters, may further clarify the clinical significance of non-coronary myocardial injury after SAH. With advances in artificial intelligence and machine learning, multidimensional data-fusion models may eventually support precision risk assessment, but such tools should undergo rigorous external validation before clinical implementation.

## Data Availability

The raw data supporting the conclusions of this article will be made available by the authors, without undue reservation.

## Glossary

GOS: Glasgow Outcome Scale
CAD: Coronary Artery Disease
RONSD: Right Optic Nerve Sheath Diameter
GCS: Glasgow Coma Scale
SBP: Systolic Blood Pressure
WBC: White Blood Cell Count
HGB: Hemoglobin
CREA: Creatinine
hsCRP: High-sensitivity C-Reactive Protein
DDHS: D-Dimer
aSAH: Aneurysmal Subarachnoid Hemorrhage
IQR: Interquartile Range
CI: Confidence Interval
ROC: Receiver Operating Characteristic
DCA: Decision Curve Analysis
hs-cTnT: High-sensitivity Cardiac Troponin T
NPE: Neurogenic Pulmonary Edema
LONSD: Left Optic Nerve Sheath Diameter
HR: Heart Rate
DBP: Diastolic Blood Pressure
NEUT: Neutrophil Percentage
PLT: Platelet Count
PCT: Procalcitonin
TC: Takotsubo Cardiomyopathy
CT: Computed Tomography
OR: Odds Ratio
VIF: Variance Inflation Factor
AUC: Area Under the Curve
T: Body Temperature
